# A novel role for interferon regulatory factor 1 (*IRF1*) in regulation of bone metabolism

**DOI:** 10.1111/jcmm.12327

**Published:** 2014-06-20

**Authors:** Sandra Salem, Chan Gao, Ailian Li, Huifen Wang, Loan Nguyen-Yamamoto, David Goltzman, Janet E Henderson, Philippe Gros

**Affiliations:** a Department of Biochemistry, McGill UniversityMontreal, QC, Canada; b Department of Medicine, McGill UniversityMontreal, QC, Canada; c Bone Engineering Labs, Research Institute of the McGill University Health CentreMontreal, QC, Canada; d Department of Physiology, McGill UniversityMontreal, QC, Canada; e Department of Surgery, McGill UniversityMontreal, QC, Canada

**Keywords:** bone development, mesenchymal cell differentiation, osteoclast differentiation, bone resorption, knockout mouse

## Abstract

Increased risk of bone fractures is observed in patients with chronic inflammatory conditions, such as inflammatory bowel disease and rheumatoid arthritis. Members of the Interferon Response Factor family of transcriptional regulators, IRF1 and IRF8, have been identified as genetic risk factors for several chronic inflammatory and autoimmune diseases. We have investigated a potential role for the *Irf1* gene in bone metabolism. Here, we report that *Irf1*^*−/−*^mutant mice show altered bone morphology in association with altered trabecular bone architecture and increased cortical thickness and cellularity. *Ex vivo* studies on cells derived from bone marrow stimulated with Rank ligand revealed an increase in size and resorptive activity of tartrate-resistant acid-positive cells from *Irf1*^*−/−*^ mutant mice compared with wild-type control mice. *Irf1* deficiency was also associated with decreased proliferation of bone marrow-derived osteoblast precursors *ex vivo*, concomitant with increased mineralization activity compared with control cells. We show that Irf1 plays a role in bone metabolism and suggest that Irf1 regulates the maturation and activity of osteoclasts and osteoblasts. The altered bone phenotype of *Irf1*^*−/−*^ mutants is strikingly similar to that of *Stat1*^*−/−*^ mice, suggesting that the two interacting proteins play a critical enabling role in the common regulation of these two cell lineages.

## Introduction

Interferon regulatory factor-1 (IRF1) is a transcriptional activator belonging to the IRF family of transcription factors. This family consists of nine members (IRF1–9) that play critical roles in immune and inflammatory responses and in host defences against infections, as well as regulating the development and apoptosis of immune cells [1] IRF proteins share significant sequence similarity in the amino terminal portions (aa1–115) that contain their defining DNA-binding domain (DBD). IRF1 also has a carboxyl terminal domain that serves as a recruitment module for other transcription factors including other members of the IRF (*e.g*. IRF8), or ETS (*e.g*. PU.1) families, to activate or repress gene expression [2].

Interferon regulatory factor-1 is expressed broadly and is required for the ontogeny and function of the lymphoid and myeloid compartments of the immune system, including T lymphocytes, natural killer (NK) cells and macrophages [2]. *Irf1*^*−/−*^ mice are immuno-deficient, and susceptible to infections. They carry lineage-specific defects in thymocyte development characterized by a marked reduction in CD8^+^ T cells [3], a decrease in NK cell numbers with associated impaired cytolytic activity [4] as well as reduced numbers of CD8α^+^ dendritic cells (DCs) concomitant with a skewed differentiation of CD11b^+^ cells towards plasmacytoid DCs (pDCs) [5]. IRF1 is an important regulator of the myeloid- and lymphoid-mediated inflammatory response. Human *IRF1* has been detected as a genetic risk factor for IBD [6] and has also been associated with the incidence and severity of synovial inflammation in type II collagen-induced arthritis [7]. IRF1 mRNA and protein expression is regulated in a cell cycle–dependent fashion, and *Irf1*-deficient mouse embryo fibroblasts do not undergo DNA damage-induced apoptosis [8]. IRF1 also acts as a negative growth regulator, and loss of IRF1 function has been associated with transformation in several cancers, including pre-leukaemic myelo-dysplastic syndromes [9]. IRF8 is another member of the IRF family of transcription factors that is expressed exclusively in cells of the immune system, including B and T lymphocytes, macrophages and DCs [10]. Like *Irf1*^*−/−*^ mutant mice, *Irf8*^*−/−*^ mice lack both CD8^+^ DCs and pDCs, have impaired T-cell function and defective IL12-dependent Th1 polarization of the early immune response [11,12]. In humans, loss of function mutations in *IRF8* cause a severe form of DC immunodeficiency [13]. At the molecular level, IRF8 and IRF1 synergize in the IFNγ-dependent activation of intrinsic macrophage anti-microbial defences (iNOS, gp91^phox^, p67^phox^, caspase-1, Cox2), of inflammatory cytokines activating early immune response (IL12-p40, IL18, RANTES, TNF-α) and of genes affecting maturation of myeloid (DCs) and lymphoid (NK, CD8^+^ T cells) cells [14–16]. Several of these direct IRF8/IRF1 downstream target genes play critical roles in bone metabolism by promoting (Cox-2, TNF-α) or inhibiting (IL12, IL18, RANTES) bone resorption [17–22].

Recent genome-wide association studies have pointed to a role for both *IRF1* and *IRF8* in genetically complex and heterogeneous human inflammatory diseases, including ulcerative colitis and Crohn's disease [6,23–25]. Increased risk of bone fractures occurs in patients with chronic inflammatory conditions such as IBD, in which IRF1 and IRF8 have been identified as genetic risks [26,27]. A role for Irf8 in bone metabolism was recently demonstrated when *Irf8*^*−/−*^ mice displayed bone defects because of enhanced osteoclastogenesis, resulting in increased bone resorption and reduced trabecular bone density [28]. Considering the critical and complementary roles played by Irf8 and Irf1 in myeloid lineage cell maturation and in the aetiology of chronic inflammatory diseases, we investigated a possible role of Irf1 in bone metabolism. In this study, we show that *Irf1*^*−/−*^ mice have an abnormal bone phenotype as a result of altered development and function of both bone-resorbing osteoclasts and bone-forming osteoblasts, thereby uncoupling their tightly regulated interaction. Our results suggest a novel role for *Irf1* as a regulator of bone metabolism in mice.

## Materials and methods

### Western blotting

Interferon regulatory factor-1 protein was evaluated in cultures of osteoclasts that were derived from bone marrow cells of 8-week-old wild-type (WT) mice as described above and in MC3T3 osteoblast-like cells. Cells were washed with cold PBS and lysed for 30 min. on ice in 0.5 ml of lysis buffer (50 mM Tris-HCl pH 7.5, 150 mM NaCl, 1% Triton X-100, 0.1% SDS) supplemented with protease inhibitors. Cell lysates were clarified by centrifugation (15 min., 4°C, 13,000 g). Whole cell extracts (50 μg) were separated on 12% SDS-polyacrylamide gel and transferred by electro-blotting onto 0.45 μm Protran BA 85 membrane (GE Healthcare, Buckinghamshire, England). Equal loading of extract and transfer of protein were verified by staining with Ponceau S red (Sigma-Aldrich, St. Louis, MO, USA). Blots were incubated with rabbit anti-IRF1M-20 antibody (1:250; Santa Cruz, Santa Cruz, CA, USA) in TBST (10 mM Tris-HCl pH 8, 150 mM NaCl, 0.05% Tween 20) plus 5% skim milk (16 hrs at 4°C), followed by washing and incubation with an anti-rabbit secondary antibody conjugated to HRP (1:20,000; GE Healthcare). Chemiluminescence was used for the detection of immune complexes on the immunoblot (SuperSignal West Pico, Thermo Scientific, Freiburg, Germany).

### Analysis of bone phenotypes

Eight-week-old male *Irf1*^*−/−*^mutant mice and WT C57BL/6J controls were used for all experiments, except when otherwise indicated. *Irf1*^*−/−*^ mice were purchased as breeding pairs from the Jackson Laboratory (Bar Harbor, ME, USA). The mutant stock name is B6.129S2-Irf1<tm1Mak>/J. Male mice were used for all experiments to avoid age-related variability that is greater in females than in males for the C57BL/6J strain [29]. Femurs were dissected at necropsy and fixed in 70% ethanol; they were then subjected to radiological imaging by using a Faxitron® MX-20 instrument at a voltage of 21 kV and current of 550 μA. Micro-computed tomographic imaging (Micro-CT) was used to investigate trabecular bone micro-architecture and was performed with a Skyscan 1072 instrument. Bone mineral density of femurs was performed with the GE Lunar Piximus instrument. All the above imaging was performed at the Centre for Bone and Periodontal Research at McGill University. Qualitative and quantitative Micro-CT analysis of cortical and trabecular bone was performed at the Bone Engineering Labs at the Research Institute-McGill University Health Centre by using a Skyscan 1172 instrument (Bruker Corp., Billerica, MA, USA). Analysis of cortical bone was performed on a 1.1-mm-long region of the diaphysis extending from the distal end of third femoral trochanter. Analysis of trabecular bone was performed separately on growth plate proximal and growth plate distal regions each measuring 1.65 mm in length. Radiology performed on seven mice per strain (*n* = 7) and significance determined by using a Student's *t*-test.

### Histology and histomorphometry

Femurs from 8-week-old or 4-month-old male mice were isolated and fixed overnight in 4% paraformaldehyde, then rinsed three times in PBS. Tissues were embedded in poly(methyl methacrylate) (PMMA) and 4–6 μm sections were prepared. Tissue section were then stained with haematoxylin and eosin for general morphology, with von Kossa/Toluidine blue to distinguish mineralized from soft tissue, for tartrate-resistant acid phosphatase (TRAP) activity to identify osteoclasts, and alkaline phosphatase (ALP) activity for hypertrophic chondrocytes and osteoblasts. Images were captured by using a Leica DMR microscope and histomorphometric data were obtained by using Bioquant Nova Prime image analysis software and ImageJ software. Histology and Histomorphometry performed on five mice per strain (*n* = 5).

### Calcein double labelling

Eight-week-old male mice were given an intra-peritoneal injection of 100 μl/20 g of calcein solution (0.25% calcein-AM and 2% NaHCO_3_ dissolved in 0.15 M NaCl) at day 10 and day 3 prior to euthanasia on day 0. Femurs were harvested, fixed in 70% ethanol and embedded in PMMA. Sections were cut at 10 μm and examined by fluorescence microscopy. Mineral apposition rate (MAR) and bone formation rate (BFR) were calculated by using ImageJ where mineralizing surface (MS) = dLS + sLS/2; dLS is the double-labelling surface perimeter and sLS is the single-labelling perimeter. MAR = distance between the 2 labels/time (7 days) and BFR = MAR × (MS/BS); BS is the total bone surface. Calcein double labelling was performed on five mice per strain (*n* = 5).

### MSC proliferation and osteoblast differentiation

Whole bone marrow was harvested from 8-week-old male mice and adherent mesenchymal stromal cells (MSCs) isolated as described previously [30]. The soft tissue and the knee ends of the femora and tibiae were removed under aseptic conditions before placing the bones in Eppendorf tubes and centrifuging at low speed to dislodge the marrow. For proliferation studies, cells were seeded at a density of 10,000 cells/cm^2^, cultured in DMEM and the cells trypsinized and counted with a haemocytometer on days 3, 6 and 9. For differentiation studies, the cells were seeded in individual 3-cm tissue culture dishes at a density of 50,000 cells/cm^2^. Osteoblast differentiation was initiated by cultivation in osteogenic DMEM medium (Wisent, Montreal, Quebec, Canada) supplemented with 10% FBS, 50 mg/ml ascorbic acid and 10 mM β-glycerophosphate for 10 and 15 days, changing the medium every 3 days. Cultures were terminated by fixation in 4% paraformaldehyde and rinsing with PBS. Alkaline phosphatase activity was detected by immersing the dishes in ALP reaction buffer (0.6 mg/ml Fast Red Violet LB salt; Sigma-Aldrich), 0.1 M Tris-HCl pH 8.3, 0.1 mg/ml naphthol AS-MX phosphate disodium salt and 0.4% *N*,*N*-dimethylformamide for 15 min. at 20°C. Dishes were then stained with 1% silver nitrate solution (von Kossa stain) under ultraviolet light for 20 min. to identify mineral deposits, washed three times with dH_2_O and images acquired by using a light microscope. MSC proliferation and differentiation experiments were performed on three mice per strain (*n* = 3). Significance was determined by using a Student's *t*-test.

### Reverse transcription and quantitative real-time PCR

Total RNA was isolated from cells cultured in osteogenic medium as described above by using commercial Trizol reagent (Invitrogen, Carlsbad, CA, USA). Aliquots of 1 μg total RNA were used to generate cDNA by using reverse transcriptase (MMLV-RT; Invitrogen) and priming with a combination of oligo dT and random hexamers. For quantitative real-time PCR (RT-PCR), all reactions were carried out by using PerfeCTa® SYBR® Green Supermix, ROX (Quanta BioSciences Inc., Gaithersburg, MD, USA) with a melting temperature of 95°C for 15 sec., an annealing temperature of 60°C for 15 sec. and an extension temperature of 72°C for 30 sec. (for 40–50 cycles). The following primer pairs were used for lineage-specific transcripts *Ocn*: 5′-GCCTTCATGTCCAAGCAGGA-3′ and 5′-GCGCCGGAGTCTGTTCACTA-3′, *Opn*: 5′-GATGATGATGACGATGGAGACC-3′ and 5′-CGACTGTAGGGACGAT TGGAG-3′, *Mgp*: 5′-CCTGTGCTACGAATCTCACGAA-3′ and 5′-TCGCAGGC CTCTCTGTTGAT-3′, *Rankl*: 5′-CACAGCGCTTCTCAGGAGCT-3′ and 5′-CAT CCAACCATGAGCCTTCC-3′. The level of mRNA expression of each target gene was normalized by using *Gapdh* as an internal house keeping control gene. RNA expression performed on three mice per strain (*n* = 3).

### *In vitro* osteoclast differentiation

Whole bone marrow harvested from the femurs of 8-week-old male *Irf1*^*−/−*^ mutant and WT control mice was plated in tissue culture dishes containing α-MEM medium (Wisent) supplemented with 10% heat-inactivated foetal bovine serum (FBS; Wisent) and 10% L-cell conditioned medium (LCCM) [31], and cultured for 5 days with one change of medium at day 2. The immature bone marrow-derived macrophages (BMDMs) obtained were then used to initiate osteoclast cultures. Briefly, plastic-adherent BMDMs were harvested by scraping, and seeded in 6-well tissue culture dishes at a density of 2.5 × 10^4^ cells/well, and cultured in α-MEM supplemented with 10% FBS, 5% LCCM and 50–100 ng/ml RANKL (Peprotech, Rocky Hill, NJ, USA) for 6 days, changing medium every 2 days, until multi-nucleated cells (>3 nuclei)appeared. TRAP staining was performed with the acid phosphatase leukocyte kit (Sigma-Aldrich) according to the manufacturer's instructions and the number of TRAP-positive cells was recorded. Osteoclast differentiation was performed with three mice per strain (*n* = 3). Significance was determined by using a Student's *t*-test.

### Resorption pit assay

Bone marrow-derived macrophages were produced as described above, and seeded in substrate-coated 6-well tissue culture plates (Osteo Assay surface plates; Corning, NY, USA) at a density of 2 × 10^4^ cells/well and incubated for 6 days in α-MEM medium containing 10% FBS, 5% LCCM and 50–100 ng/ml RANKL to induce osteoclast differentiation, with medium changes every 2 days. After 6 days, 10% bleach was added to the Osteo Assay surface plates for 5 min. at 20°C to terminate the assay, followed by extensive washing with distilled water. Resorption pits were identified and counted by using a microscope and the relative resorption area calculated by using ImageJ software. Resorption pit assay was performed with three mice per strain (*n* = 3). Significance was determined by using a Student's *t*-test.

### Three-point bending assay

Biomechanical testing was performed on femurs of 8-week-old male *Irf1*^*−/−*^ mice and WT littermates at the Centre for Bone and Periodontal Research of McGill University (Montreal, QC, Canada) by using a three-point bending test on a Mach-1TM Micromechanical Systems device. Failure loads were analysed by using the Mach-1TM Motion and Analysis software (version 3.0.2, Bio SyntechCanada, Quebec, Canada). A load-displacement curve was generated by using this software to measure biomechanical parameters, including stiffness (N/mm), ultimate force (N), ultimate displacement (μm) and work to failure (N*mm). Biomechanical testing was performed on five mice per strain (*n* = 5). Significance was determined by using a Student's *t*-test.

## Results

To investigate a possible role of Irf1 in bone metabolism, we first investigated Irf1 protein expression in bone cells. Immunoblotting studies performed with the MC3T3 osteoblast-like cell line and primary osteoclasts derived from bone marrow *ex vivo* identified Irf1 protein expression in both osteoblast and osteoclast cell lineages (Fig. S1). A possible role of Irf1 in bone metabolism in general, and in bone cell function in particular, was then investigated by comparative analyses of tissues and cells from normal mice and from mice carrying a null mutation at *Irf1* (*Irf1*^*−/−*^).

### *Irf1* deficiency results in altered bone morphology and mineral content

**Fig. 1:**
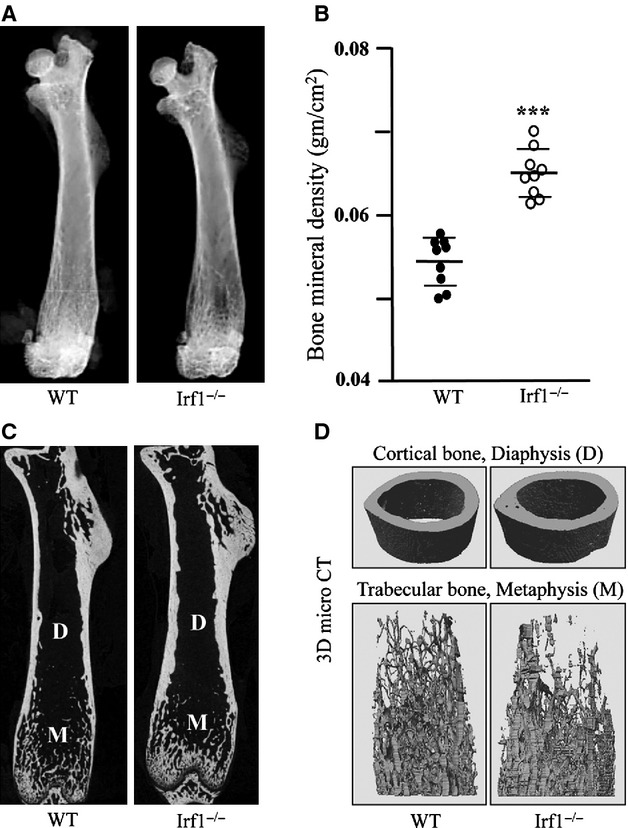
Radiological analysis of femurs from control and *Irf1*^*−/−*^ mice. High-resolution X-ray (**A**), bone mineral density (**B**) 2D (**C**) and 3D (**D**) micro-CT from representative 8-week-old wild-type (Bl6) and mutant (Irf1−/−) are shown. B: ***P < 0.001.

Radiological imaging (Fig. 1) was first used to screen for differences in the appendicular skeletons of WT and *Irf1*^*−/−*^mutant mice. High-resolution X-rays (Fig. 1A) revealed shorter femurs with increased distal radiolucency in the *Irf1*^*−/−*^ mice. Bone mineral density (Fig. 1B) was anomalously increased in the mutant compared with WT mice. Micro-CT analyses (Fig. 1C and D) confirmed a reduction in trabecular bone in the proximal (removed from the growth plate), but not distal (adjacent to the growth plate), metaphysis (M) of *Irf1*^*−/−*^ mice and increased cortical bone width in the diaphysis (D). Quantitative micro-CT analyses (Table 1) of bone structure and composition were performed separately on trabecular bone adjacent to, and removed from, the growth plate and on cortical bone. There were no significant differences in quantitative data in trabecular bone adjacent to the growth plate except for an increase in the number of closed pores, which represent spaces occupied by marrow, in *Irf1*^*−/−*^compared with WT mice. In contrast, the total amount of bone (BV/TV) and the number of trabeculae (Tb.N) were reduced while trabecular thickness (Tb.Th) was increased, and the spaces between trabeculae increased (Tb.Sp) in the mutant compared with WT mice. The total volume (TV) of cortical bone was increased, but the surface (BS/TV) decreased in the mutant compared with WT mice, confirming the apparent increase in cortical width seen in the 2D micro-CT sections (Fig. 1). The number and volume of closed pores, which represent osteocytes or vascular channels in cortical bone, were also significantly increased in the mutant mice. The altered structure model index values in mutant mice (Table 1) are indicative of osteopenia in trabecular bone and of increased connectivity in cortical bone.

**Table 1:** Quantitative micro-CT

Parameter (unit)	Trabecular bone adjacent to GP			Trabecular bone away from GP			Cortical bone in diaphysis		
			
	WT	*Irf1*^*−/−*^	*P*	WT	*Irf1*^*−/−*^	*P*	WT	*Irf1*^*−/−*^	*P*
TV	4.31	4.43	NS	2.97	2.74	NS	1.10	1.20	0.009
BV/TV (%)	16.1	16.2	NS	5.75	3.41	0.008	98.9	99.0	NS
BS/TV (mm^−1^)	11.5	11.2	NS	4.83	2.89	0.001	13.1	11.9	0.002
Tb.Sp (mm^−1^)	0.22	0.25	NS	0.34	0.61	0.008	–	–	–
Tb.N (mm^−1^)	2.63	2.55	NS	0.93	0.50	0.004	–	–	–
Po.N (cl)	23.5	76.5	0.001	2.00	4.75	NS	150	429	0.002
SMI			NS	2.68	3.46	0.025	0.16	−1.52	0.002

TV, total volume; BV/TV, bone volume/total volume; BS/TV, bone surface/total volume; Tb.Sp, trabecular separation; Tb.N, trabecular number; Po.N (cl), number of closed pores; SMI, structure model index.

### Histological evidence of reduced trabecular and increased cortical bone in *Irf1*^*−/−*^ mice

**Fig. 2:**
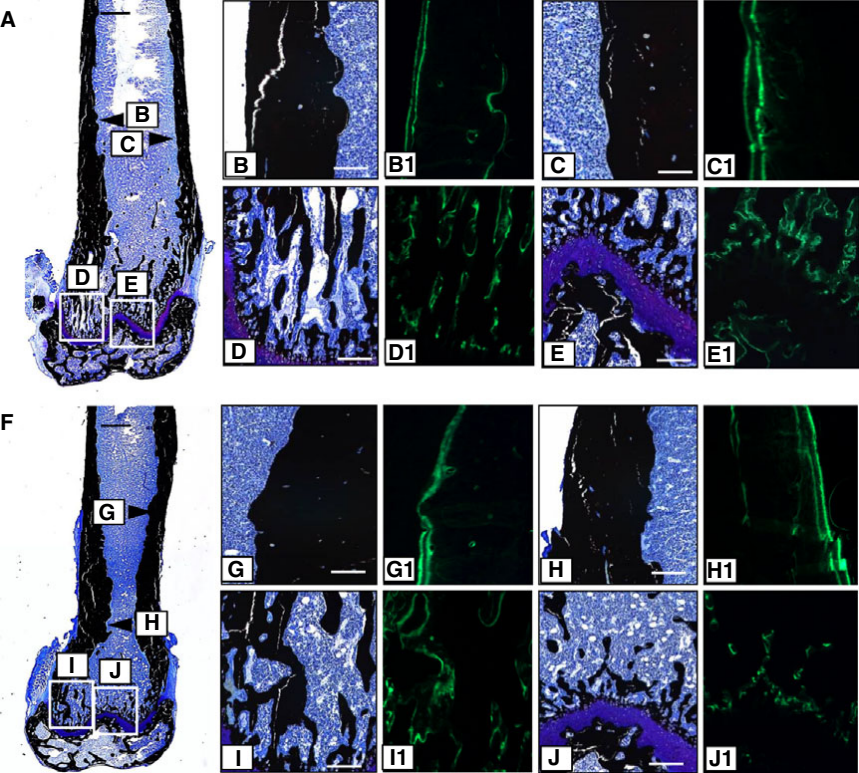
Histological analysis of bone apposition in control and *Irf1*^*−/−*^ mice. Representative B6 (**A**–**E**) and *Irf1*^*−/−*^ (**F**–**J**) bone was sectioned at 10 μm and imaged with a fluorescence microscope before staining with Von Kossa/Toluidine blue and re-imaging. Boxed areas (**A** and **F**) identify the regions shown at higher magnification (**B**, **C**, **G**, **H**) with the corresponding fluorescence images of calcein labelling. Arrowheads indicate the regions of cortical bone shown at higher magnification (**D**, **E**, **I**, **J**) with the corresponding fluorescence images. Scale bars represent 500 μm (**A** and **F**) and 200 μm (**B**–**E**, **G**–**J**).

**Fig. 3:**
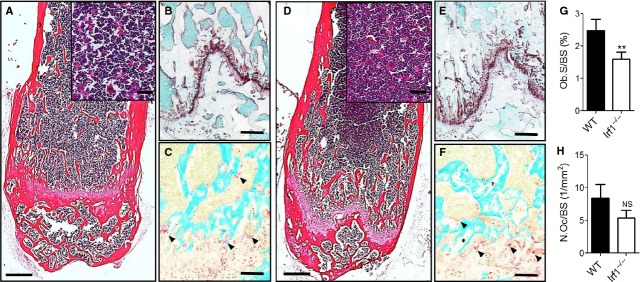
Histochemical staining of femurs from control and *Irf1*^*−/−*^ mice. Five-micron sections of decalcified bone from representative B6 (**A**) and *Irf1*^*−/−*^ (**D**) mice were stained with haematoxylin and eosin (**A** and **D**) for bone marrow (inset). Five-micron sections of un-decalcified bone were stained for alkaline phosphatase activity (brown) in hypertrophic chondrocytes and osteoblasts (**B** and **E**) or for tartrate-resistant acid phosphatase activity (arrowheads) in osteoclasts (**C** and **F**). Scale bars represent 500 μm (**A** and **D**) and 50 μm (inset) or 200 μm (**B**, **C**, **E**, **F**). Bone histomorphometric measurements of osteoblast surface per bone surface (Ob. S/BS; **G**) and osteoclast number per bone surface (N. Oc/BS; **H**).

The long bones of immature 2-month-old mice continue to grow in length and in width. Thick sections (10 μm) of un-decalcified femurs were examined under fluorescence microscopy, to evaluate calcein labelling and stained with von Kossa and toluidine blue to assess bone micro-structure (Fig. 2). In von Kossa-stained sections harvested from the mid-diaphysis, the distal femurs were longer and cortical bone thinner in representative WT (Fig. 2A) compared with *Irf1*^*−/−*^ mutant (Fig. 2F) mice. Calcein (green) was deposited at the endosteal and periosteal surfaces of cortical bone with little apparent difference between WT (Fig. 2B1–E1) and *Irf1*^*−/−*^ (Fig. 2G1–J1) femurs. The orderly deposition of calcein along trabeculae arising from the growth plate in WT mice (Fig. 2D1 and E1) was reflected in the well-defined structures aligned with the longitudinal axis of the bone (Fig. 2D and E). In contrast, von Kossa-stained sections (Fig. 2I and J) and calcein-labelled (Fig. 2I1–J1) sub-chondral bone in *Irf1*^*−/−*^ mutant mice were both disorganized and diminished. Adjacent thin (5 μm) sections stained with haematoxylin and eosin revealed hypercellular bone marrow in some of the mutant mice (Fig. 3D inset),decreased alkaline phosphatase staining in Irf1^−/−^ mice (Fig. 3B and E) and no difference between WT and *Irf1*^*−/−*^mice in TRAP activity (Fig. 3C and F arrows) in osteoclasts. Bone histomorphometric measurements confirmed a decrease in osteoblast surface in *Irf1*^*−/−*^, and no difference in osteoclast number compared with WT (Fig. 3G and H).

### *Irf1* deficiency results in increased MSC proliferation and differentiation *ex vivo*

**Fig. 4:**
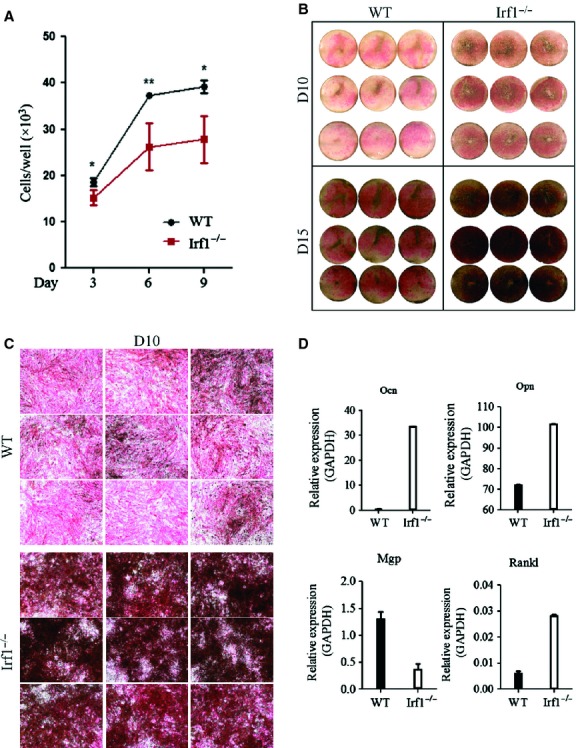
Altered osteoblast function in *Irf1*^*−/−*^ mice. (**A**) Marrow stromal cells (MSCs) isolated from 8-week-old male mice were maintained in medium and cells were counted at days 3, 6 and 9. Results represented as means ± SD, *n* = 4. **P < 0.05. (**B**) Bone MSCs were isolated from 8-week-old male *Irf1*^*−/−*^ mice and WT controls and maintained in osteoblast differentiation medium consisting of ascorbic acid and β-glycerophosphate for 10 or 15 days. Cultures were fixed in 4% paraformaldehyde and stained *in situ* for ALP activity and with silver nitrate (von Kossa) to detect mineralized nodules. (**C**) Microscopic images of mineralized nodules (three independent experiments; *n* = 3 mice/group). (**D**) Quantitative real-time PCR analysis of mRNA expression of Osteocalcin (*Ocn*), Osteopontin (*Opn*), Matrix gla protein (*Mgp*), Receptor activator of NFκB ligand (*Rankl*) in cultured osteoblasts (at day 6). Expression of test genes is relative to *Gapdh*, which was used as an internal control (mean of three experiments ± SD).

**Fig. 5:**
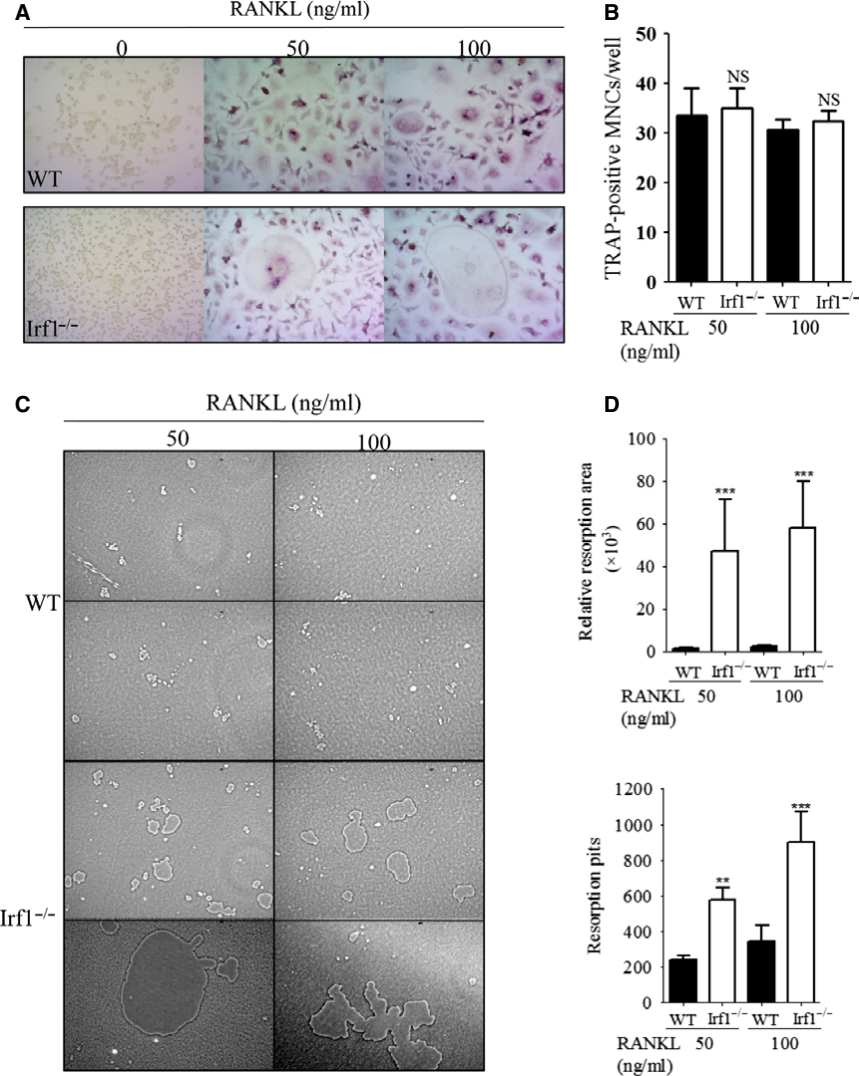
Enhanced osteoclast activity and bone resorption in *Irf1*^*−/−*^ mice. (**A**) Bone marrow-derived macrophages (BMMs) from 8-week-old *Irf1*^*−/−*^ mice and WT controls were stimulated with RANKL in the presence of M-CSF to produce osteoclasts. After 7 days, TRAP staining was performed. TRAP-positive cells appear as large, multi-nucleated cells (MNCs). (**B**) The number of TRAP-positive MNCs containing at least three nuclei was quantitated. (**C**) BMMs were plated on OsteoAssay plates coated with inorganic synthetic followed by stimulation with RANKL for 7 days to induce formation of resorption pits. (**D**) The number of resorption pits, and the total relative resorption area were counted by using Image J software. Results represented as means from nine independent experiments (±SD). ***P < 0.0001, **P < 0.001. NS, no statistical significance.

To uncover the cellular cause of the altered bone phenotype, we examined the effect of loss of function of *Irf1* on the proliferation of MSCs and their differentiation into mature osteoblasts *ex vivo*, by using alkaline phosphatase activity (Fig. 4, pink) as an early marker and von Kossa staining as an index of mineral deposition at a later stage (Fig. 4, black). MSCs isolated from *Irf1*^*−/−*^mutant mouse bone marrow also proliferated more slowly than those isolated from bone marrow of WT mice (Fig. 4A). Interestingly, however, MSCs from *Irf1*^*−/−*^mutant mice differentiated more rapidly and deposited significantly more mineral (black) than cells harvested from WT mice cultured under the same conditions (Fig. 4B and C). Total cellular RNA isolated from the cultures on Day 6 after induction of differentiation was used to monitor expression of osteoblast lineage markers. Consistent with the more rapid differentiation and increased mineral deposition seen in the *Irf1*^*−/−*^cultures, significantly higher levels of expression of mRNAs encoding Osteocalcin (*Ocn*) and Osteopontin (*Opn*), and lower levels of Matrix Gla protein (*Mgp*), were detected in *Irf1*^*−/−*^ compared with WT cells (Fig. 5D). *Irf1*^*−/−*^cells also showed a sixfold increase in expression of the mRNA for the osteoclastogenesis inducer Rank ligand (*Rankl*), when compared with WT cells, suggesting a stronger osteoblast-directed signal for bone destruction by osteoclasts in *Irf1*^*−/−*^mice.

### *Irf1* deficiency results in enhanced osteoclast activity *ex vivo*

We then examined the effect of *Irf1* loss of function on osteoclast formation and osteoclast function *ex vivo* (Fig. 5). Primary BMDMs were cultured for 7 days in the presence of RANK ligand (tested at 50 and 100 ng/ml) to induce osteoclast formation *ex vivo*. These experiments did not reveal an effect of loss of *Irf1* on the total number of TRAP-positive multi-nucleated cells formed under these conditions, although the cells appeared larger in size than those from WT mice (Fig. 5A and B). The activity of the WT and *Irf1* mutant osteoclasts was assessed by their capacity to form resorption pits on a synthetic calcium phosphate bone surface. TRAP-positive multi-nucleated cells derived from *Irf1*^*−/−*^ mouse marrow formed significantly more pits with a greater area than the equivalent cells derived from WT mouse marrow. Similar results were seen with both concentrations of RANK ligand tested (Fig. 5C and D). These results suggested that *Irf1* regulates osteoclast maturation and/or activity, which could impact on bone metabolism *in vivo*. In agreement with these findings, we detected higher serum levels of calcium and phosphorus in *Irf1* mutants than in controls; however, no differences in serum TRAP or osteocalcin (Fig. S2A–D).

### *Irf1* deficiency leads to stiffer long bones

**Fig. 6:**
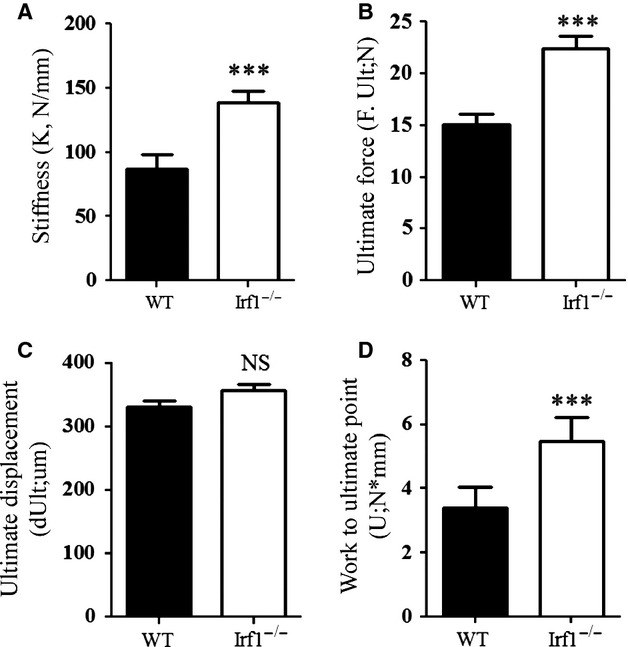
Biomechanical testing reveals stronger bones in Irf1^−/−^ mice. Biomechanical testing by a three-point bending assay was performed and analysis demonstrated stiffness (**A**), force (**B**), ultimate displacement (d.Ult; **C**) and ultimate force to point (**D**); *n* = 5. Results represented as means (±SD). ***P < 0.0001. NS, no statistical significance.

The increase in trabecular and cortical thickness, enhanced osteoblast differentiation and higher mineralization led us to examine the strength of the long bones from age-matched *Irf1*^*−/−*^ mice and WT littermates (Fig. 6). Biomechanical testing by a three-point bending assay revealed that femurs from *Irf1*^*−/−*^ mice were stiffer (K) than those of WT mice, with a larger ultimate force (N) and more work to failure (U) (Fig. 6A–D). These results suggest that *Irf1* deficiency leads to stronger bones, because of the enhanced osteoblast differentiation and mineral deposition.

## Discussion

This study provides evidence for a novel role for *Irf1* in regulating long bone development, which is critically dependent on the orderly and co-ordinated activity of osteoblasts and osteoclasts [32]. *Irf1*^*−/−*^ mice exhibit alterations in femoral morphology, micro-structure and bone cell activity. X-ray and quantitative micro-CT analyses revealed shorter femurs with altered trabecular architecture resulting from fewer, wider trabeculae in the mutant compared with WT mice. The changes in bone micro-structure were confirmed by histological analyses of un-decalcified bone, which showed no apparent differences in ALP activity in osteoblasts or TRAP activity in osteoclasts lining the trabeculae. The reduction in bone length and altered trabecular bone architecture are therefore attributed to changes in the turnover of cartilage to bone in the growth plates. Cortical bone width and cellularity were also increased in the *Irf1*^*−/−*^ mice, which accounted for the increase in BMD and enhanced biomechanical strength of their long bones compared with those of WT control. Histological analysis confirmed the increase in cortical bone width in the mutant mice, but with no apparent change in the rate of deposition (calcein labelling) suggesting it represents an adaptive mechanism to maintain mechanical strength in the face of decreased and disorganized trabecular bone architecture [33].

Bone remodelling is achieved through a precise balance between the activity of bone-resorbing osteoclasts and bone-forming osteoblasts. The proliferation of osteoblast progenitor cells and their differentiation into mineralizing osteoblasts is tightly regulated during bone development and in the adult skeleton undergoing remodelling [34]. In previous *in vitro* studies, *Irf1* mRNA was detected in proliferating precursor cells and in differentiated osteoblasts depositing mineral [35]. It was therefore not surprising that Irf1 protein was present in cultures of MC3T3 mouse osteoblast-like cells (Fig. S1). We now show that cultures of MSCs isolated from the bone marrow of *Irf1*^*−/−*^mice proliferate more slowly and differentiate into mineralizing osteoblasts more rapidly than those isolated from WT mice. These observations suggest that Irf1 may act as a negative regulator of osteoblast function, possibly by maintaining the precursor cells in a proliferative, poorly differentiated state with no capacity for matrix mineralization. Consistent with this conjecture was the significant up-regulation of *Ocn*, and down-regulation of *Matrix gla protein* mRNA in differentiating cultures of *Irf1*^*−/−*^cells. OCN and MGP are non-collagenous proteins that promote and inhibit tissue mineralization respectively. Of additional interest was the increased expression of OPN in the mutant osteoblasts, which has an undefined role in tissue mineralization, but is believed to anchor osteoclasts to bone matrix during the remodelling cycle. Cultures of *Irf1*^*−/−*^ cells also expressed sixfold more *Rankl* mRNA than WT cells. RANKL is a cell surface protein on osteoblasts that binds to its receptor RANK on osteoclast precursors and stimulates their differentiation and activity.

Osteoclasts arise from the same myeloid progenitor cells that give rise to tissue macrophages and DCs, as well as circulating monocytes [36]. These cells express Irf1 and Irf8, and require both factors for their maturation and for the function of fully differentiated cells [2,5,37] (Fig. S1). In agreement with the *in vivo* TRAP staining data, there appeared to be no difference in the number of TRAP-positive cells in cultures of *Irf1*^*−/−*^ and WT control bone marrow cells stimulated with RANKL to induce osteoclastogenesis. The TRAP-positive cells from the mutant mice were, however, larger and resorbed more pits of a larger size in synthetic bone *ex vivo* than the cells harvested from WT control mice. A similar correlation between the size of cultured TRAP-positive cells and their *ex vivo* resorptive capacity has been shown for cells isolated from rabbits [38] and from genetically modified mice with synovitis and excessive sub-chondral bone resorption [39]. The later situation reflects that seen in patients with rheumatoid arthritis, where large mononuclear and multi-nucleate TRAP-positive cells in the synovium are thought to contribute to both cartilage and bone destruction [40]. The fact that serum calcium and phosphorous were elevated in the mutant compared with WT mice (Fig. S2), in association with a reduction in trabecular bone, suggests that osteoclast activity was indeed elevated in *Irf1*^*−/−*^mice. Taken together, the data indicate that Irf1 may suppress the activity of differentiated osteoclasts and/or maintain them in a less differentiated state.

The increase in osteoclast activity is similar to that seen in *Irf8*^*−/−*^ mice, which lack the dimerization partner for Irf1 [28]. This highlights key roles for Irf1 and Irf8 in development and function of the myeloid lineage in general, and of osteoclasts in particular. *Irf1*^*−/−*^ mutant mice show immune defects in the myeloid and lymphoid cell compartments, including impaired Th1 immune response, decreased pro-inflammatory cytokine production and susceptibility to intracellular pathogens [11,12,14–16]. Pro-inflammatory cytokines have been shown to contribute to regulation of bone metabolism [19]. Two important cytokines, IL-12 and IL-18, are expressed in osteoblasts and act to decrease osteoclastogenesis by inhibiting Rankl production and other aspects of osteoclast formation and activity [19–21]. These pro-inflammatory anti-osteoclastogenic cytokines are not produced by *Irf1*^*−/−*^mice [41]. When translated to the *in vivo* situation, the significant differences in *ex vivo* growth and activity of cells of both the osteoblast and osteoclast lineages would ultimately impact on the biomechanical properties of the long bones of *Irf1*^*−/−*^ mice. The functional consequence of altered bone cell activity was evident in a three-point bending test where the force applied to the mid-diaphysis that was required to break the bone was significantly higher in the mutant compared with animals. The data suggest that the balance between enhanced osteoblast and osteoclast function was tipped in favour of osteogenesis.

The bone phenotype of *Irf1*^*−/−*^ mice is strikingly similar to that of mice bearing a null allele at *Stat1* (*Stat1*^*−/−*^; signal transducer and activator of transcription 1). Stat1 is a transcriptional regulator that physically and functionally interacts with Irf1 in the regulation of gene expression downstream interferon gamma signalling [42,43]. Similar to Irf1 deficiency, *Stat1*^*−/−*^ mutant mice show an increase in bone mass, in cortical bone thickness and in bone mineral density, because of increased osteoblast function despite having excessive osteoclastogenesis [44]. Stat1 plays an important regulatory role in both osteoclast and osteoblast differentiation and function. In osteoclast precursor cells, Stat1 is required for IFN-β-dependent dampening of RANKL-induced expression of c-fos, a transcription factor essential for osteoclastogenesis [45]. Stat1 is also required for IFN-γ-dependent inhibition of RANKL expression by activated T cells [46,47]. In osteoblasts, Stat1 has been shown to inhibit the function of Runx2, a transcription factor that plays a central role in bone formation [44]. The net increase in bone formation and resulting bone mass suggests that *in vivo*, the regulatory function of Stat1 in osteoblasts dominates over its inhibitory function in osteoclasts. Irf1 is a well-known transcriptional activator of IFN-β genes [48], which are critical for regulating the skeletal system by inhibiting osteoclastogenesis [49]. Thus, we propose that absence of Irf1 would eliminate this regulatory function and enhance osteoclastogenesis. Although the mechanism of Irf1 negative regulation of osteoblast differentiation and function remains to be elucidated, it is tempting to speculate that it may involve the formation of Irf1/Stat1 heterodimers [42].

The current study identifies a novel role for Irf1 in bone metabolism during development. Further experiments will be required to identify the transcriptional targets of Irf1 that regulate the maturation and activity of cells of the osteoclast and osteoblast lineages. Likewise, it will be interesting to investigate a possible role of IRF1 and of its heterodimerization partner IRF8 in human disorders characterized by osteopenia, such as osteoporosis. Although genome-wide association studies have identified positive genetic associations with markers in the proximity of the *IRF1* (5q31.1) and *IRF8* (16q24.1) genes [50,51], a role for these genes and their targets in clinical osteoporosis needs to be formally tested.

## Supporting information

**Figure S1.** Detection of Irf1 expression in bone cells. Total cell lysates of osteoclasts derived from bone marrow (BM) cells of B6 mice and MC3T3 cells were analysed by SDS-PAGE and immunoblotting with anti-IRF1M-20 antibody. Irf1 expression was compared to bone marrow-derived macrophages (BMDMs) stimulated with Interferon-γ (IFNγ) which was used as a positive control and HEK 293T cells used as a negative control.

**Figure S2.** Serum biochemistry. Serum from *Irf1*^*−/−*^ mice and WT littermates was analysed for calcium, phosphorous, TRAP and Osteocalcin levels.

## References

[1] Barnes B, Lubyova B, Pitha PM (2002). On the role of IRF in host defense. J Interferon Cytokine Res.

[2] Harada H, Fujita T, Miyamoto M (1989). Structurally similar but functionally distinct factors, IRF-1 and IRF-2, bind to the same regulatory elements of IFN and IFN-inducible genes. Cell.

[3] Penninger JM, Sirard C, Mittrucker HW (1997). The interferon regulatory transcription factor IRF-1 controls positive and negative selection of CD8+ thymocytes. Immunity.

[4] Taki S, Sato T, Ogasawara K (1997). Multistage regulation of Th1-type immune responses by the transcription factor IRF-1. Immunity.

[5] Gabriele L, Fragale A, Borghi P (2006). IRF-1 deficiency skews the differentiation of dendritic cells toward plasmacytoid and tolerogenic features. J Leukoc Biol.

[6] Jostins L, Ripke S, Weersma RK (2012). Host-microbe interactions have shaped the genetic architecture of inflammatory bowel disease. Nature.

[7] Tada Y, Ho A, Matsuyama T (1997). Reduced incidence and severity of antigen-induced autoimmune diseases in mice lacking interferon regulatory factor-1. J Exp Med.

[8] Tanaka N, Ishihara M, Kitagawa M (1994). Cellular commitment to oncogene-induced transformation or apoptosis is dependent on the transcription factor IRF-1. Cell.

[9] Willman CL, Sever CE, Pallavicini MG (1993). Deletion of IRF-1, mapping to chromosome 5q31.1, in human leukemia and preleukemic myelodysplasia. Science.

[10] Eisenbeis CF, Singh H, Storb U (1995). Pip, a novel IRF family member, is a lymphoid-specific, PU.1-dependent transcriptional activator. Genes Dev.

[11] Meraro D, Hashmueli S, Koren B (1999). Protein-protein and DNA-protein interactions affect the activity of lymphoid-specific IFN regulatory factors. J Immunol.

[12] Tamura T, Ozato K (2002). ICSBP/IRF-8: its regulatory roles in the development of myeloid cells. J Interferon Cytokine Res.

[13] Hambleton S, Salem S, Bustamante J (2011). IRF8 mutations and human dendritic-cell immunodeficiency. N Engl J Med.

[14] Xiong H, Zhu C, Li H (2003). Complex formation of the interferon (IFN) consensus sequence-binding protein with IRF-1 is essential for murine macrophage IFN-gamma-induced iNOS gene expression. J Biol Chem.

[15] Eklund EA, Jalava A, Kakar R (1998). PU.1, interferon regulatory factor 1, and interferon consensus sequence-binding protein cooperate to increase gp91(phox) expression. J Biol Chem.

[16] Liu J, Guan X, Tamura T (2004). Synergistic activation of interleukin-12 p35 gene transcription by interferon regulatory factor-1 and interferon consensus sequence-binding protein. J Biol Chem.

[17] Zhang X, Schwarz EM, Young DA (2002). Cyclooxygenase-2 regulates mesenchymal cell differentiation into the osteoblast lineage and is critically involved in bone repair. J Clin Invest.

[18] Lam J, Takeshita S, Barker JE (2000). TNF-alpha induces osteoclastogenesis by direct stimulation of macrophages exposed to permissive levels of RANK ligand. J Clin Invest.

[19] Lorenzo J, Horowitz M, Choi Y (2008). Osteoimmunology: interactions of the bone and immune system. Endocr Rev.

[20] Amcheslavsky A, Bar-Shavit Z (2006). Interleukin (IL)-12 mediates the anti-osteoclastogenic activity of CpG-oligodeoxynucleotides. J Cell Physiol.

[21] Horwood NJ, Udagawa N, Elliott J (1998). Interleukin 18 inhibits osteoclast formation *via* T cell production of granulocyte macrophage colony-stimulating factor. J Clin Invest.

[22] Wintges K, Beil FT, Albers J (2013). Impaired bone formation and increased osteoclastogenesis in mice lacking chemokine (C-C motif) ligand 5 (Ccl5). J Bone Miner Res.

[23] Franke A, McGovern DP, Barrett JC (2010). Genome-wide meta-analysis increases to 71 the number of confirmed Crohn's disease susceptibility loci. Nat Genet.

[24] Barrett JC, Hansoul S, Nicolae DL (2008). Genome-wide association defines more than 30 distinct susceptibility loci for Crohn's disease. Nat Genet.

[25] Elding H, Lau W, Swallow DM (2011). Dissecting the genetics of complex inheritance: linkage disequilibrium mapping provides insight into Crohn disease. Am J Hum Genet.

[26] Ali T, Lam D, Bronze MS (2009). Osteoporosis in inflammatory bowel disease. Am J Med.

[27] Lin CL, Moniz C, Chambers TJ (1996). Colitis causes bone loss in rats through suppression of bone formation. Gastroenterology.

[28] Zhao B, Takami M, Yamada A (2009). Interferon regulatory factor-8 regulates bone metabolism by suppressing osteoclastogenesis. Nat Med.

[29] Glatt V, Canalis E, Stadmeyer L (2007). Age-related changes in trabecular architecture differ in female and male C57BL/6J mice. J Bone Miner Res.

[30] Valverde-Franco G, Liu H, Davidson D (2004). Defective bone mineralization and osteopenia in young adult FGFR3-/- mice. Hum Mol Genet.

[31] Fleit HB, Rabinovitch M (1981). Interferon induction in marrow-derived macrophages: regulation by L cell conditioned medium. J Cell Physiol.

[32] Marks SC, Popoff SN (1988). Bone cell biology: the regulation of development, structure, and function in the skeleton. Am J Anat.

[33] Turner CH, Hsieh YF, Muller R (2000). Genetic regulation of cortical and trabecular bone strength and microstructure in inbred strains of mice. J Bone Miner Res.

[34] Harada S, Rodan GA (2003). Control of osteoblast function and regulation of bone mass. Nature.

[35] Lynch MP, Capparelli C, Stein JL (1998). Apoptosis during bone-like tissue development *in vitro*. J Cell Biochem.

[36] Fujikawa Y, Quinn JM, Sabokbar A (1996). The human osteoclast precursor circulates in the monocyte fraction. Endocrinology.

[37] Kamijo R, Harada H, Matsuyama T (1994). Requirement for transcription factor IRF-1 in NO synthase induction in macrophages. Science.

[38] Lees RL, Sabharwal VK, Heersche JN (2001). Resorptive state and cell size influence intracellular pH regulation in rabbit osteoclasts cultured on collagen-hydroxyapatite films. Bone.

[39] Doody KM, Bussieres-Marmen S, Li A (2012). T cell protein tyrosine phosphatase deficiency results in spontaneous synovitis and subchondral bone resorption in mice. Arthritis Rheum.

[40] Tsuboi H, Matsui Y, Hayashida K (2003). Tartrate resistant acid phosphatase (TRAP) positive cells in rheumatoid synovium may induce the destruction of articular cartilage. Ann Rheum Dis.

[41] Lohoff M, Ferrick D, Mittrucker HW (1997). Interferon regulatory factor-1 is required for a T helper 1 immune response *in vivo*. Immunity.

[42] Chatterjee-Kishore M, Wright KL, Ting JP (2000). How Stat1 mediates constitutive gene expression: a complex of unphosphorylated Stat1 and IRF1 supports transcription of the LMP2 gene. EMBO J.

[43] Ramsauer K, Farlik M, Zupkovitz G (2007). Distinct modes of action applied by transcription factors STAT1 and IRF1 to initiate transcription of the IFN-gamma-inducible gbp2 gene. Proc Natl Acad Sci USA.

[44] Kim S, Koga T, Isobe M (2003). Stat1 functions as a cytoplasmic attenuator of Runx2 in the transcriptional program of osteoblast differentiation. Genes Dev.

[45] Stark GR, Kerr IM, Williams BR (1998). How cells respond to interferons. Annu Rev Biochem.

[46] Kong YY, Feige U, Sarosi I (1999). Activated T cells regulate bone loss and joint destruction in adjuvant arthritis through osteoprotegerin ligand. Nature.

[47] Takayanagi H, Ogasawara K, Hida S (2000). T-cell-mediated regulation of osteoclastogenesis by signalling cross-talk between RANKL and IFN-gamma. Nature.

[48] Taniguchi T, Ogasawara K, Takaoka A (2001). IRF family of transcription factors as regulators of host defense. Annu Rev Immunol.

[49] Takayanagi H, Kim S, Matsuo K (2002). RANKL maintains bone homeostasis through c-Fos-dependent induction of interferon-beta. Nature.

[50] Hsu YH, Zillikens MC, Wilson SG (2010). An integration of genome-wide association study and gene expression profiling to prioritize the discovery of novel susceptibility Loci for osteoporosis-related traits. PLoS Genet.

[51] Rivadeneira F, Styrkarsdottir U, Estrada K (2009). Twenty bone-mineral-density loci identified by large-scale meta-analysis of genome-wide association studies. Nat Genet.

