# Supplementary figures and images for: A novel role for interferon regulatory factor 1 (IRF1) in regulation of bone metabolism
Source: J Cell Mol Med. 2014 Jun 20;18(8):1588–98. doi: 10.1111/jcmm.12327 (PMC4152406; doi:10.1111/jcmm.12327)

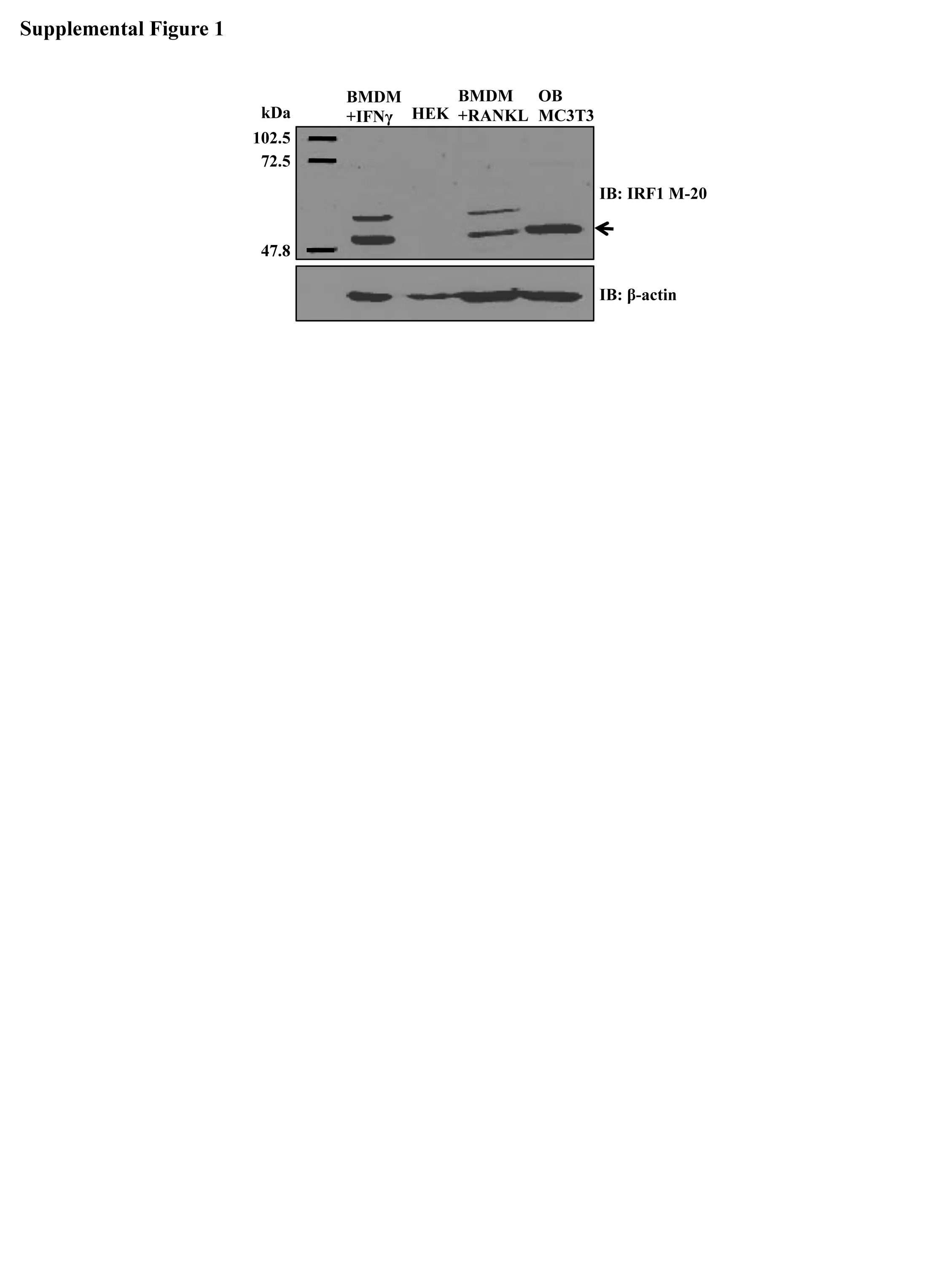

Supplement: Supplementary file 1 — Figure S1. Detection of Irf1 expression in bone cells. Total cell lysates of osteoclasts derived from bone marrow (BM) cells of B6 mice and MC3T3 cells were analysed by SDS-PAGE and immunoblotting with anti-IRF1M-20 antibody. Irf1 expression was compared to bone marrow-derived macrophages (BMDMs) stimulated with Interferon-γ (IFNγ) which was used as a positive control and HEK 293T cells used as a negative control. [file jcmm0018-1588-SD1.tiff]

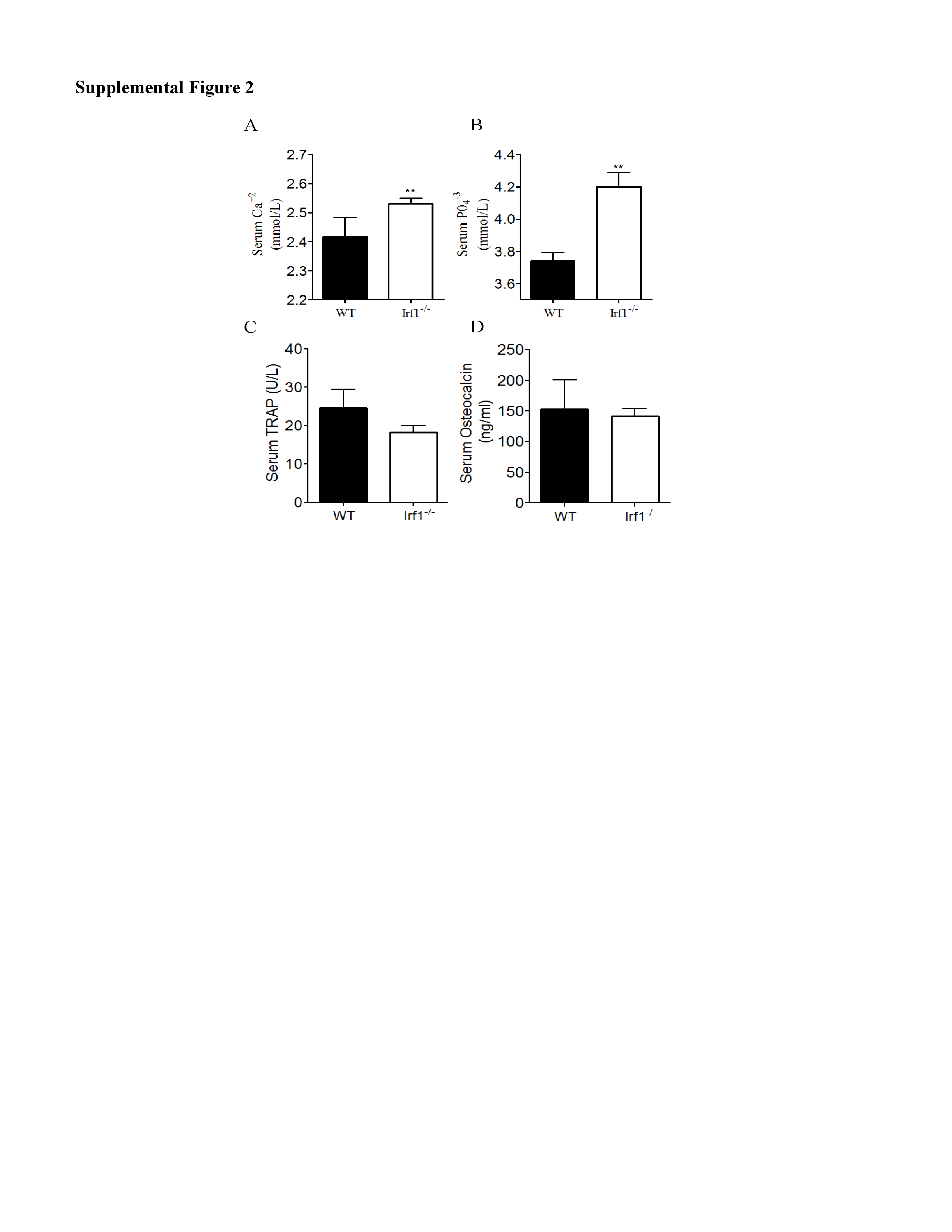

Supplement: Supplementary file 2 — Figure S2. Serum biochemistry. Serum from Irf1−/− mice and WT littermates was analysed for calcium, phosphorous, TRAP and Osteocalcin levels. [file jcmm0018-1588-SD2.tiff]
